# Supplementary material for: Psychosocial factors and hospitalisations for COVID-19: Prospective cohort study based on a community sample
Source: Brain Behav Immun. 2020 Oct;89:569–78. doi: 10.1016/j.bbi.2020.06.021 (PMC7297693; doi:10.1016/j.bbi.2020.06.021)
Supplement: Supplementary data 1 [file mmc1.docx]

**Supplemental Table 1. Analyses of apparent known risk factors (based on clinical studies) for COVID-19 hospitalisation in UK Biobank (N=431,052)**

|  | **All COVID-19 cases**  **(N=908)** | **COVID-19 cases known to have been hospitalised (‘origin’=1) (N=751)** | **COVID-19 cases known to have been hospitalised (‘origin’=1) *and* with >=2 tests conducted (N=445)** |
| --- | --- | --- | --- |
|  |  |  |  |
| *Age* |  |  |  |
| 40-49 | Ref (1.0) | Ref (1.0) | Ref (1.0) |
| 50-59 | 0.69 (0.57, 0.83) | 0.77 (0.63, 0.95) | 0.81 (0.62, 1.05) |
| 60+ | 1.09 (0.93, 1.27) | 1.16 (0.97, 1.38) | 1.17 (0.83, 1.48) |
| Per decade increase | 1.15 (1.06, 1.25) | 1.18 (1.08, 1.29) | 1.17 (1.04, 1.32) |
|  |  |  |  |
| *Sex* |  |  |  |
| Female | Ref (1.0) | Ref (1.0) | Ref (1.0) |
| Male | 1.53 (1.35, 1.75) | 1.56 (1.35, 1.81) | 1.55 (1.28, 1.87) |
|  |  |  |  |
| *Ethnicity* |  |  |  |
| White | Ref (1.0) | Ref (1.0) | Ref (1.0) |
| Non-white | 2.65 (2.20, 3.20) | 2.45 (1.98, 3.03) | 3.12 (2.42, 4.01) |
|  |  |  |  |
| *Long-standing illness* |  |  |  |
| No | Ref (1.0) |  |  |
| Yes | 2.06 (1.80, 2.35) | 2.03 (1.75, 2.04) | 2.26 (1.87, 2.73) |
|  |  |  |  |

**Supplemental Table 2. Odds ratios (95% CI) for the relation of socioeconomic factors with COVID-19 hospitalisation *– based on complete data***

|  | **Case N /Risk N^1^** | **Adjustments** | | | | | |
| --- | --- | --- | --- | --- | --- | --- | --- |
|  |  | **Age & sex** | **Age, sex & ethnicity** | **Age, sex, ethnicity & comorbidity^1^** | **Age, sex, ethnicity & lifestyle factors^2^** | **Age, sex, ethnicity & psychological factors^3^** | **Adjusted for all covariates** |
| **Educational attainment** |  |  |  |  |  |  |  |
| University Degree | 90/55905 | 1.0 (ref) | 1.0 (ref) | 1.0 (ref) | 1.0 (ref) | 1.0 (ref) | 1.0 (ref) |
| Other qualifications | 144/77597 | 1.17 (0.89, 1.52) | 1.19 (0.91, 1.55) | 1.15 (0.88, 1.49) | 1.03 (0.79, 1.34) | 1.06 (0.81, 1.39) | 0.94 (0.71, 1.24) |
| No qualifications | 54/19237 | 1.70 (1.20, 2.41) | 1.75 (1.23, 2.48) | 1.60 (1.12, 2.27) | 1.28 (0.89, 1.84) | 1.30 (0.89, 1.90) | 1.01 (0.68, 1.49) |
| P for trend |  | 0.005 | 0.003 | 0.015 | 0.245 | 0.214 | 0.945 |
|  |  |  |  |  |  |  |  |
| **Annual household income** |  |  |  |  |  |  |  |
| <£18,000 | 68/26578 | 1.90 (1.31 2.77) | 1.79 (1.23, 2.60) | 1.63 (1.12, 2.38) | 1.41 (0.96, 2.08) | 1.41 (0.95, 2.09) | 1.15 (0.77, 1.73) |
| £18,000-£30,999 | 71/34301 | 1.52 (1.06, 2.20) | 1.47 (1.02, 2.11) | 1.41 (0.98, 2.03) | 1.28 (0.89, 1.85) | 1.28 (0.88, 1.86) | 1.15 (0.79, 1.68) |
| £31,000-£51,999 | 60/36526 | 1.19 (0.82, 1.72) | 1.16 (0.80, 1.68) | 1.14 (0.79, 1.65) | 1.07 (0.74, 1.56) | 1.09 (0.75, 1.57) | 1.02 (0.70, 1.48) |
| ≥£52,000 | 54/38368 | 1.0 (ref) | 1.0 (ref) | 1.0 (ref) | 1.0 (ref) | 1.0 (ref) | 1.0 (ref) |
| P for trend |  | <0.0001 | 0.001 | 0.006 | 0.053 | 0.060 | 0.401 |
|  |  |  |  |  |  |  |  |
| **Townsend Deprivation Index** | |  |  |  |  |  |  |
| 1 (low) | 69/48529 | 1.0 (ref) | 1.0 (ref) | 1.0 (ref) | 1.0 (ref) | 1.0 (ref) | 1.0 (ref) |
| 2 | 102/53718 | 1.34 (0.99, 1.82) | 1.31 (0.97, 1.78) | 1.29 (0.95, 1.75) | 1.24 (0.91, 1.68) | 1.28 (0.94, 1.74) | 1.22 (0.89, 1.65) |
| 3 | 121/51407 | 1.70 (1.26, 2.29) | 1.55 (1.14, 2.09) | 1.46 (1.08, 1.97) | 1.28 (0.94, 1.75) | 1.41 (1.04, 1.92) | 1.20 (0.87, 1.63) |
| P for trend |  | <0.0001 | 0.005 | 0.016 | 0.130 | 0.030 | 0.297 |
|  |  |  |  |  |  |  |  |
| **Occupational classification** |  |  |  |  |  |  |  |
| Managers, senior officials, etc | 154/76773 | 1.0 (ref) | 1.0 (ref) | 1.0 (ref) | 1.0 (ref) | 1.0 (ref) | 1.0 (ref) |
| Administrative, secretarial, etc | 42/29676 | 0.71 (0.51, 1.01) | 0.71 (0.51, 1.01) | 0.70 (0.50, 0.99) | 0.65 (0.46, 0.91) | 0.64 (0.45, 0.90) | 0.59 (0.42, 0.84) |
| Personal service, sales, etc | 48/21630 | 1.12 (0.81, 1.55) | 1.08 (0.78, 1.50) | 1.04 (0.75, 1.45) | 0.90 (0.65, 1.25) | 0.86 (0.61, 1.21) | 0.75 (0.53, 1.06) |
| P for trend |  | 0.993 | 0.887 | 0.713 | 0.219 | 0.145 | 0.027 |

^1^Comorbidity includes diagnoses of vascular or heart disease, diabetes, chronic bronchitis or emphysema, asthma, and hypertension defined according to measured blood pressure and/or use of anti-hypertensive medication. ^2^ Lifestyle factors includes body mass index, smoking status, alcohol intake frequency & number of types of physical activity taken in last four weeks. ^2^ Psychological factors include psychological distress, psychiatric consultation, neuroticism, verbal and numerical reasoning, & reaction time.

**Supplemental Table 3. Odds ratios (95% CI) for the relation of psychological factors with COVID-19 hospitalisation *– based on complete data***

|  | **Case N /**  **Risk N^1^** | **Adjustments** | | | | | |
| --- | --- | --- | --- | --- | --- | --- | --- |
|  |  | **Age & sex** | **Age, sex & ethnicity** | **Age, sex, ethnicity & comorbidity^1^** | **Age, sex, ethnicity & lifestyle factors^2^** | **Age, sex, ethnicity & socioeconomic factors^3^** | **Adjusted for all covariates** |
| **Psychological distress** |  |  |  |  |  |  |  |
| 1 (low) | 149/96723 | 1.0 (ref) | 1.0 (ref) | 1.0 (ref) | 1.0 (ref.) | 1.0 (ref.) | 1.0 (ref) |
| 2 | 161/93337 | 1.14 (0.91, 1.42) | 1.15 (0.92, 1.44) | 1.12 (0.89, 1.40) | 1.09 (0.87, 1.36) | 1.13 (0.91, 1.42) | 1.07 (0.86, 1.34) |
| 3 | 113/555059 | 1.36 (1.06, 1.75) | 1.32 (1.03, 1.70) | 1.25 (0.97, 1.61) | 1.15 (0.89, 1.48) | 1.22 (0.95, 1.57) | 1.09 (0.84, 1.40) |
| P for trend |  | 0.016 | 0.028 | 0.080 | 0.272 | 0.107 | 0.501 |
| Per SD increase |  | 1.18 (1.08, 1.29) | 1.16 (1.06, 1.27) | 1.13 (1.03, 1.24) | 1.09 (1.00, 1.20) | 1.12 (1.02, 1.22) | 1.07 (0.97, 1.17) |
|  |  |  |  |  |  |  |  |
| **Psychiatric consultation** |  |  |  |  |  |  |  |
| No | 412/238768 | 1.0 (ref) | 1.0 (ref) | 1.0 (ref) | 1.0 (ref) | 1.0 (ref) | 1.0 (ref) |
| Yes | 58/26798 | 1.26 (0.96, 1.67) | 1.29 (0.98, 1.70) | 1.26 (0.95, 1.66) | 1.19 (0.90, 1.57) | 1.22 (0.92, 1.61) | 1.15 (0.87, 1.52) |
|  |  |  |  |  |  |  |  |
| **Neuroticism** |  |  |  |  |  |  |  |
| 1 (low) | 119/69458 | 1.0 (ref) | 1.0 (ref) | 1.0 (ref) | 1.0 (ref) | 1.0 (ref) | 1.0 (ref) |
| 2 | 191/109358 | 1.03 (0.82, 1.30) | 1.05 (0.83, 1.32) | 1.03 (0.81, 1.29) | 1.02 (0.81, 1.28) | 1.03 (0.82, 1.29) | 1.01 (0.80, 1.27) |
| 3 | 157/85968 | 1.09 (0.85, 1.39) | 1.11 (0.87, 1.41) | 1.06 (0.83, 1.36) | 1.03 (0.81, 1.32) | 1.06 (0.83, 1.35) | 1.00 (0.79, 1.28) |
| P for trend |  | 0.491 | 0.395 | 0.612 | 0.804 | 0.660 | 0.982 |
| Per SD increase |  | 1.03 (0.94, 1.13) | 1.04 (0.94, 1.14) | 1.02 0.93, 1.12) | 1.00 (0.91, 1.10) | 1.01 (0.92, 1.11) | 0.99 (0.90 1.09) |
|  |  |  |  |  |  |  |  |
| **Verbal numerical reasoning** |  |  |  |  |  |  |  |
| 1 (low) | 81/26374 | 2.52 (1.83, 3.47) | 2.09 (1.50, 2.93) | 2.03 (1.45, 2.84) | 1.86 (1.33, 2.62) | 2.06 (1.44, 2.96) | 1.98 (1.38, 2.84) |
| 2 | 86/41404 | 1.70 (1.24, 2.34) | 1.61 (1.17, 2.21) | 1.59 (1.16, 2.18) | 1.53 (1.11, 2.11) | 1.60 (1.16, 2.21) | 1.58 (1.14, 2.18) |
| 3 | 70/57112 | 1.0 (ref) | 1.0 (ref) | 1.0 (ref) | 1.0 (ref) | 1.0 (ref) | 1.0 (ref) |
| P for trend |  | <0.0001 | <0.0001 | <0.0001 | <0.0001 | <0.001 | <0.0001 |
| Per SD decrease |  | 1.46 (1.27, 1.66) | 1.34 (1.17, 1.54) | 1.32 (1.15, 1.52) | 1.27 (1.10, 1.46) | 1.33 (1.15, 1.55) | 1.31 (1.12, 1.52) |
|  |  |  |  |  |  |  |  |
| **Reaction time** |  |  |  |  |  |  |  |
| 1 (low) | 164/99395 | 1.0 (ref) | 1.0 (ref) | 1.0 (ref) | 1.0 (ref) | 1.0 (ref) | 1.0 (ref) |
| 2 | 155/88713 | 1.09 (0.87 1.36) | 1.05 (0.84, 1.31) | 1.04 (0.84, 1.31) | 1.03 (0.82, 1.29) | 1.03 (0.82, 1.29) | 1.02 (0.81, 1.28) |
| 3 | 148/76914 | 1.21 (0.96, 1.53) | 1.10 (0.87, 1.39) | 1.09 (0.86, 1.37) | 1.05 (0.83, 1.33) | 1.04 (0.82, 1.31) | 1.02 (0.80, 1.29) |
| P for trend |  | 0.104 | 0.424 | 0.496 | 0.659 | 0.759 | 0.869 |
| Per SD increase |  | 1.16 (1.06, 1.27) | 1.10 (1.01, 1.21) | 1.09 (1.00, 1.20) | 1.08 (0.99, 1.19) | 1.07 (0.98, 1.17) | 1.06 (0.97, 1.17) |
|  |  |  |  |  |  |  |  |

^1^ Comorbidity includes diagnoses of vascular or heart disease, diabetes, chronic bronchitis or emphysema, asthma, and hypertension defined according to measured blood pressure and/or use of anti-hypertensive drugs. ^2^ Lifestyle factors included body mass index, smoking status, alcohol intake frequency & number of types of physical activity taken in last four weeks. ^3^ Socioeconomic factors included occupational classification, highest educational attainment, Townsend deprivation index, & household income before tax

**Supplementary Table 4. Odds ratios (95% CI) for the relation of socioeconomic factors with COVID-19 hospitalisation *– impact of adjusting for biomarkers***

|  |  | **Adjustments** | | |
| --- | --- | --- | --- | --- |
|  | **Case N /Risk N^1^** | **Age, sex & ethnicity** | **Age, sex, ethnicity & biomarker^2^** | **All covariates^3^** |
| **Educational attainment** |  | N=420502 | N=301981 | N=108462 |
| Degree | 229/137717 | 1.0 (ref) | 1.0 (ref) | 1.0 (ref) |
| Other qualifications | 406/214337 | 1.19 (1.01, 1.41) | 1.15 (0.93, 1.41) | 0.94 (0.66, 1.33) |
| No qualifications | 241/70003 | 2.07 (1.71, 2.50) | 1.82 (1.43, 2.31) | 1.10 (0.68, 1.77) |
| P for trend |  | <0.0001 | <0.0001 | 0.802 |
|  |  |  |  |  |
| **Annual household income** |  | N=363175 | N=261825 | N=96562 |
| <£18,000 | 241/81207 | 1.89 (1.51, 2.35) | 1.47 (1.12, 1.93) | 1.03 (0.62, 1.72) |
| £18,000-£30,999 | 179/92461 | 1.27 (1.01, 1.60) | 1.09 (0.83, 1.44) | 1.04 (0.65, 1.68) |
| £31,000-£51,999 | 167/95454 | 1.17 (0.94, 1.47) | 1.10 (0.84, 1.49) | 1.13 (0.72, 1.78) |
| ≥£52,000 | 141/95097 | 1.0 (ref) | 1.0 (ref) | 1.0 (ref) |
| P for trend |  | <0.0001 | 0.007 | 0.988 |
|  |  |  |  |  |
| **Neighbourhood deprivation** |  | N=427986 | N=301418 | N=108898 |
| 1 (low) | 205/143483 | 1.0 (ref) | 1.0 (ref) | 1.0 (ref) |
| 2 | 267/143548 | 1.29 (1.07, 1.55) | 1.14 (0.91, 1.42) | 1.10 (0.76, 1.58) |
| 3 | 436/143517 | 1.97 (1.66, 2.34) | 1.58 (1.28, 1.96) | 0.97 (0.66, 1.42) |
| P for trend |  | <0.0001 | <0.0001 | 0.840 |
|  |  |  |  |  |
| **Occupational classification** |  | N=307262 | N=221854 | N=91388 |
| Managers, senior officials, etc | 324/175637 | 1.0 (ref) | 1.0 (ref) | 1.0 (ref) |
| Administrative, secretarial, etc | 94/74137 | 0.69 (0.55, 0.87) | 0.65 (0.49, 0.87) | 0.48 (0.30,0.77) |
| Personal service, sales, etc | 149/58915 | 1.30 (1.07, 1.59) | 1.28 (1.01, 1.64) | 0.75 (0.48, 1.15) |
| P for trend |  | 0.091 | 0.223 | 0.054 |

^1^ Numbers based on unadjusted model. ^2^Biomarkers included FEV1, and blood concentrations of c-reactive protein, HbA1c, and HDL cholesterol. ^3^Multivariate model included age, sex, ethnicity, diagnoses of vascular or heart disease, diabetes, chronic bronchitis or emphysema, asthma, hypertension defined according to measured blood pressure and/or use of anti-hypertensive drug, body mass index, smoking status, alcohol intake frequency, number of types of physical activity taken in last four week, psychological distress, psychiatric consultation, reasoning, reaction time, FEV1, and blood concentrations of c-reactive protein, HbA1c and HDL cholesterol.

**Supplementary Table 5. Odds ratios (95% CI) for the relation of psychological factors with COVID-19 hospitalisation – *impact of adjusting for biomarkers***

|  | **Case N /**  **Risk N^1^** | **Adjustments** | | |
| --- | --- | --- | --- | --- |
|  |  | **Age, sex & ethnicity** | **Age, sex, ethnicity & biomarkers^2^** | **All covariates^3^** |
| **Psychological distress** |  | N=383655 | N=273998 | N=179391 |
| 1 (low) | 267/153504 | 1.0 (ref) | 1.0 (ref) | 1.0 (ref) |
| 2 | 291/140200 | 1.29 (1.09, 1.53) | 1.16 0.94, 1,43) | 1.04 (0.79, 1.36) |
| 3 | 224/91205 | 1.51 (1.26, 1.81) | 1.28 (1.01, 1,61) | 0.98 (0.71, 1.34) |
| P for trend |  | <0.0001 | 0.033 | 0.931 |
| Per SD increase |  | 1.19 (1.12, 1.26) | 1.12 (1.04, 1.22) | 1.04 (0.93, 1.17) |
|  |  |  |  |  |
| **Psychiatric consultation** |  | N=426823 | N=303561 | N=194162 |
| No | 751/379080 | 1.0 (ref) | 1.0 (ref) | 1.0 (ref) |
| Yes | 140/487739 | 1.51 (1.26, 1.81) | 1.44 (1.15, 1.82) | 1.27 (0.91, 1.76) |
|  |  |  |  |  |
| **Neuroticism** |  | N=424212 | N=302071 | N=193565 |
| 1 (low) | 224/106910 | 1.0 (ref) | 1.0 (ref) | 1.0 (ref) |
| 2 | 345/174705 | 1.03 (0.87, 1.22) | 0.98 (0.79, 1.22) | 1.03 (0.78, 1.37) |
| 3 | 319/144092 | 1.21 (1.02, 1.44) | 1.19 (0.96, 1.48) | 1.06 (0.78, 1.42) |
| P for trend |  | 0.023 | 0.088 | 0.725 |
| Per SD increase |  | 1.08 (1.01, 1.16) | 1.08 (0.99 1.17) | 0.99 (0.88, 1.11) |
|  |  |  |  |  |
| **Verbal numerical reasoning** |  | N=174581 | N=122752 | N=89129 |
| 1 (low) | 152/43988 | 2.31 (1.77, 3.02) | 2.35 (1.68, 3.30) | 2.08 (1.33, 3.28) |
| 2 | 115/58446 | 1.45 (1.10, 1.90) | 1.51 (1.07, 2.13) | 1.67 (1.12, 2.49) |
| 3 | 96/72833 | 1.0 (ref) | 1.0 (ref) | 1.0 (ref) |
| P for trend |  | <0.0001 | <0.0001 | 0.001 |
| Per SD decrease |  | 1.37 (1.23, 1.53) | 1.37 (1.19, 1.58) | 1.33 (1.10, 1.61) |
|  |  |  |  |  |
| **Reaction time** |  | N=424432 | N=302492 | N=193832 |
| 1 (low) | 262/140934 | 1.0 (ref) | 1.0 (ref) | 1.0 (ref) |
| 2 | 274/141575 | 1.00 (0.84, 1.19) | 1.01 (0.81, 1.25) | 1.09 (0.83, 1.42) |
| 3 | 345/143368 | 1.16 (0.98, 1.37) | 1.07 (0.87, 1.33) | 1.05 (0.79, 1.40) |
| P for trend |  | 0.078 | 0.505 | 0.724 |
| Per SD increase |  | 1.07(1.01, 1.14) | 1.06 (0.98, 1.15) | 1.08 (0.96, 1.21) |
|  |  |  |  |  |

^1^ Numbers based on age & sex adjusted model. ^2^ Biomarkers included FEV1, and blood concentrations of c-reactive protein, HbA1c, and HDL cholesterol. ^3^Multivariate model included age, sex, ethnicity, diagnoses of vascular or heart disease, diabetes, chronic bronchitis or emphysema, asthma, hypertension defined according to measured blood pressure and/or use of anti-hypertensive drug, body mass index, smoking status, alcohol intake frequency, number of types of physical activity taken in last four week, occupational classification, educational attainment, Townsend deprivation index, household income before tax, FEV1, and blood concentrations of c-reactive protein, HbA1c and HDL cholesterol.
